# Non-pharmacological treatment of hereditary spastic paraplegia: a systematic review

**DOI:** 10.1007/s10072-023-07200-1

**Published:** 2023-11-16

**Authors:** Simona Maccora, Angelo Torrente, Vincenzo Di Stefano, Antonino Lupica, Salvatore Iacono, Laura Pilati, Antonia Pignolo, Filippo Brighina

**Affiliations:** 1 Department of Biomedicine, Neurosciences and Advanced Diagnostics (Bi.N.D.), University of Palermo, Via del Vespro 143, 90127 Palermo, Italy; 2 Neurology Unit, ARNAS Civico di Cristina and Benfratelli Hospitals, 90127 Palermo, Italy; 3 Department of Neuroscience, “S. Giovanni di Dio” Hospital, 88900 Crotone, Italy

**Keywords:** Hereditary spastic paraplegia, Spasticity, Non-pharmacological treatment, Physiotherapy, Surgery, Non-invasive brain or spinal stimulation

## Introduction

Hereditary spastic paraplegias (HSPs) are monogenic neurological disorders characterized by slowly progressive lower extremity spasticity, bladder dysfunction, and mild proprioceptive sensory disturbances due to degeneration of long corticospinal tracts and dorsal columns [1]. Lateral corticospinal tract degeneration involves the dorsal and cervical spines [2]. According to Harding’s classification, HSP can be classified into pure and complex forms based on clinical phenotype: in pure forms, leg spasticity, and urinary symptoms such as hypertonic bladder or urinary urgency and mild decrease of vibration sensation are the unique manifestations, whereas in complex HSP, other neurological symptoms (e.g., cognitive, cerebellar, peripheral, extrapyramidal, and seizures) or non-neurological manifestations are combined with the classical syndrome [3]. More than 80 causative genes have been described so far, and inheritance patterns can be autosomal dominant (AD) in about 80% of European and North American populations, autosomal recessive (AR), X-linked, or mitochondrial, with 13–40% being sporadic [4]. The most prevalent forms of AD HSP are due to the SPAST mutation (SPG4) [5, 6], followed by the SPG3A mutation [7]. AR HSPs are rare and usually complex [4], and the most prevalent form is SPG11 [8]. X-linked and mitochondrial HSPs are very rare, often sporadic, and heterogenous [9]. Apart from clinical and inheritance pattern classification, HSPs can also be classified based on intracellular pathophysiological mechanisms such as organelle’s morphogenesis or membrane structure, bone morphogenic proteins, motor protein transportation, mitochondrial failure, axon elongation path, myelination errors, and nucleotide’s metabolism [9]. Therefore, HSPs are heterogenous disorders from a clinical, genetic, and pathophysiological perspective.

Lower limb spasticity is the main clinical feature of HSPs and is part of the upper motor neuron (UMN) syndrome [10]. An UMN syndrome is the consequence of an altered balance of excitatory and inhibitory influences on alpha motor neurons, leading to weakness, hyperexcitable spinal reflexes (withdrawal and stretch reflex), and secondary muscle abnormalities characterized by physical shortening and reduction in extensibility of soft tissues (“spastic myopathy”) [10, 11]. Spastic muscle overactivity includes dynamic manifestations such as spasms, the spastic co-contraction of antagonist muscles during voluntary activation, spastic motor overflow occurring when there is an inappropriate contraction at a distance from active movement, and static phenomena like classic spasticity which is a velocity-dependent increase in muscle tone with an increase in tendinous reflex, and spastic dystonia indicating a static increase in muscle tone without a primary triggering factor [11, 12]. Limb immobilization can promote contractures and intrinsic hypertonia, usually followed by muscle fibrosis which contributes to overactivating muscle spindles and, consequentially, the stretch reflex [13]. All these factors give rise to impaired function and quality of life in HSP patients [14].

There is no specific disease-modifying therapy for HSP, and the symptomatic approach is currently the mainstay [15]. Management of HSP should be multidisciplinary in order to achieve better control of motor symptoms such as rigidity, walking impairment, and muscle spasms, in addition to preventing skeletal deformities [16]. Pharmacological treatment takes advantage of antispastic medications such as baclofen and tizanidine which are first used to reduce spasticity, while oxybutynin is usually prescribed to control urinary urgency [1]. 4-Aminopyridine could have some role in reducing motor impairment in HSP patients [17]. Nevertheless, patients often report small benefits from conventional oral drugs. Botulinum toxin type-A (BTX-A) may help reduce spasticity and fatigue without affecting other symptoms in HSP such as depression and excessive daytime sleepiness [18, 19]. However, there could be some relief of pain in spinal cord injury patients treated with BTX-A [20], but further evidence is needed in HSP patients experiencing chronic pain. Intrathecal baclofen (ITB) can be indicated in cases of adverse effects associated with oral antispastic drugs, unsatisfactory responses to oral drugs, and wheelchair-bound patients [21]. Physical therapy and interventional/surgical approaches are other options to treat HSP patients, especially those with drug-resistant spasticity.

## Rationale

The effectiveness of non-pharmacological treatment in HSP was documented only in a few studies involving a small number of subjects, but it deserves more attention since medications can achieve better outcomes if a multidisciplinary approach is used. Furthermore, several new therapeutic approaches, such as non-invasive stimulation techniques, have been employed to control spasticity in clinical settings.

## Objectives

The aim is to investigate the use of non-pharmacological treatments in HSP, including magnetic field therapy, acupuncture, laser therapy, surgery, electric stimulation therapy, stretching, and physiotherapy.

## Methods

A systematic literature review was performed according to Preferred Reporting Items for Systematic Reviews and Meta-Analyses (PRISMA) guidelines [22]. Two large electronic bibliographic databases—PubMed and EMBASE (both last accessed on 2nd of January 2023)—were searched using the terms “Hereditary spastic paraplegia” in all fields AND “rehabilitation” OR “surgery” OR “Electric Stimulation Therapy” OR “Magnetic Field Therapy” OR “acupuncture” OR “laser therapy” OR “physiotherapy” OR “stretching” in all fields.

The search was additionally supplemented by a manual search on Google Scholar, and the references to clinically relevant reviews and abstracts published in conference proceedings were also consulted.

A language restriction was applied to evaluate only papers written in English.

The review included registries, retrospective cohorts, prospective cohort studies, and case series. Experimental and quasi-experimental studies, including clinical trials and open-label studies addressing the issue of HSP therapy, have been considered. Animal studies, conference abstracts, and review articles have been excluded.

Title and abstract screening were performed independently by two reviewers. Full-text screening was performed by two independent reviewers. When there was a disagreement, it was resolved through discussion with a third reviewer.

### Eligibility criteria

To be included in this review, papers needed to report (a) data about patients of any age “probably” or “definitely” affected by HSP if just three or all of Fink’s criteria (1996) were respectively fulfilled. (1) Slowly progressive and symmetric gait disorder characterized by lower limb spasticity and weakness, (2) family history of spastic paraparesis, (3) a corticospinal defect leading to hyperreflexia and extensor plantar responses, and (4) alternative causes of spastic paraparesis have been ruled out; this criterion must always be fulfilled to suspect HSP [23]. (b) Description of non-pharmacological treatments, including magnetic field therapy, acupuncture, laser therapy, surgery, and electric stimulation therapy.

### Outcome measures

The main outcome measure was represented by the description of the non-pharmacological treatments and the response to them in the HSP populations. Response to treatment could have been defined by clinical and/or neurophysiological measures.

Clinical response to treatment could have been a measure of motor impairment in cases of spasticity as indexed by the Modified Ashworth Scale (MAS), balance evaluated by the Berg Balance Scale (BBS) or the Timed Up and Go Test (TUG), and walking speed as assessed by the 10 Meters Walking Test (10 MWT) or the 6 Minutes Walking Test (6 MWT). Muscular strength evaluated by dynamometers, Physiological Cost Index (PCI), and gait analysis were assessed in some studies. Other clinical outcomes, such as quality of life and assessment of psychological status (e.g., anxiety or depression), were defined in a few studies. Moreover, the Spastic Paraplegia Rating Scale (SPRS) was used in only one study.

Neurophysiological evaluation was very heterogenous, ranging from peripheral nerve conduction studies (tibial nerve conduction studies, F waves) and motor-evoked potentials (MEPs) to indirect measures of spasticity such as the H-reflex or the H-reflex recovery curves to paired stimuli.

### Data extraction and synthesis methods

Data have been extracted independently by 2 reviewers and summarized in a descriptive way, considering the number of articles included in this systematic review. For each article, the following article data were extracted: name of the first author, year of publication, type of study, number of patients involved, specific HSP type, type of intervention performed, and response to the intervention.

## Results

### Study selection

The PubMed and Embase searches yielded a total of 2144 articles. The titles and abstracts were screened, and any duplicates were eliminated (98 duplicates). Two reviewers independently reviewed 2046 titles and abstracts and excluded 1929 records. Records were excluded for the following reasons: (1) animal studies; (2) review articles; (3) case reports describing single cases of HSP; (4) conference abstracts; (5) not written in English; (6) registered study protocols; (7) studies of combined pharmacological and non-pharmacological approaches in HSP; and (8) not concerning HSP therapy.

**Fig. 1:**
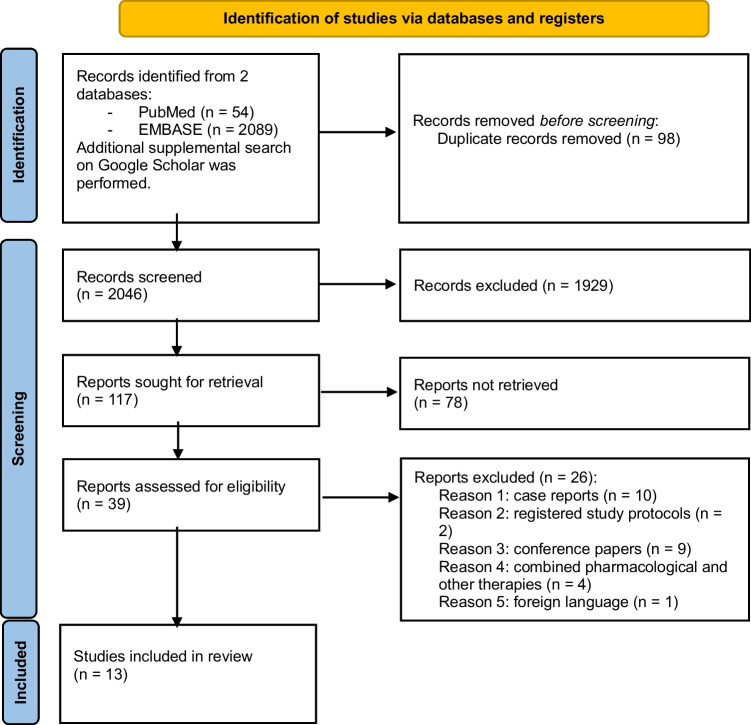
Flow diagram of the article selection process according to the Preferred Reporting Items for Systematic Reviews and Meta-Analyses (PRISMA) flowchart

One hundred and seventeen reports were evaluated, and 78 of them were not retrieved because of inappropriate study design (*n* = 13), inappropriate population (*n* = 16), inappropriate intervention (*n* = 18), inappropriate outcomes (*n* = 23), language restrictions (*n* = 2), and unavailability of full text (*n* = 7). Therefore, 39 articles underwent full-text screening. Among them, 26 were excluded because they failed to meet the inclusion criteria: 10 were case reports involving single patients, 2 were registrations of study protocols, 9 were conference abstracts, 4 used a combined pharmacological and non-pharmacological treatment, and 1 was written in Turkish. The final selection comprised 13 articles: 6 dealing with physical therapy, 2 with selective dorsal rhizotomy, and 5 discussing the possible therapeutic use of non-invasive stimulation techniques. A flow diagram of the article selection process, conforming to the PRISMA guidelines [22], is depicted in Fig. 1.

### Study characteristics

Among the selected articles, 1 was a retrospective study, 2 were case series, 4 prospective studies, 2 open-label studies, and 4 RCTs. Taken together, results obtained from 117 patients with HSP were included in the analysis, with only 37 patients presenting a genetically confirmed diagnosis (SPG3A, *n* = 3; SPG4, *n* = 20; SPG5, *n* = 1; SPG7, *n* = 8; SPG10, *n* = 1; SPG11, *n* = 1; SPG15, *n* = 1; and SPG33, *n* = 2).

## Physical therapy

Most studies were uncontrolled and involving a small number of patients. Moreover, there was no specific indication of the appropriate timing and type of physical therapy.

### Electrical stimulation

In spastic paraparesis, ankle weakness and spastic plantar flexors contribute to impaired walking. Neuromuscular electrical stimulation delivers small electrical currents to the nerves of the affected muscles, and, in the case of pairing with a motor task, it is called functional electrical stimulation (FES). FES can ameliorate visco-elastic muscle properties and inhibitory influences on the stretch reflex. Only one study evaluated the use of FES in 11 patients with spastic paraparesis, compared to 11 matched controls [24]. All patients were long-term FES users, as they had been undergoing FES for more than 6 months. It was impossible to understand how many patients did receive a precise diagnosis of HSP, although 6 of the participants had a family history of spastic paraparesis. FES could be administered as bilateral FES of the common peroneal nerve (*n* = 8) or as a preferred pattern of stimulation involving other muscle groups (*n* = 3). Outcome measures included ankle dorsiflexor torque recorded using a dynamometer, range of movement of dorsiflexion, walking speed over a 10-m walkway, a kinematic analysis using a 3D motion analysis, and the Physiological Cost Index (PCI). FES ameliorated toe clearance and maximal range of foot dorsiflexion during the swing phase and improved walking speed (*p* < 0.05) with an effect size of 0.37. Limits of this study include the absence of a naïve population of FES users, no specified genetic diagnosis, and no neurophysiological outcome or measures assessing quality of life.

### Robotic gait training

In a small study [25], a robotic gait orthosis (Lokomat, Hocoma AG, Volketswil, Switzerland) was used to treat 13 patients with pure HSP; treatment consisted of three weekly sessions for a 6-week period. Effectiveness was explored by several scales: BBS, TUG, 6 MWT, 10 MWT, MAS, PCI, Hospital Anxiety and Depression Scale (HADS), and the Short Form Health Survey 36 (SF-36) for quality of life. All the outcome measures were tested 1 week before the treatment began, at the end of the treatment, and 2 months later. Lokomat statistically ameliorated balance (BBS *p* = 0.03) and walking speed (6 MWT *p* = 0.001; 10 MWT *p* = 0.002) and improved psychological status (HADS, anxiety domain *p* = 0.002). There was also an improvement in quality of life, as assessed by multiple domains of the SF-36 scale. However, there was no reduction of spasticity as assessed by the MAS and TUG, and PCI did not change as well. The quality of this uncontrolled study is low because of no comparison with standard therapy.

### Hydrotherapy

Hydrotherapy is a conservative physical treatment taking advantage of thermal, physical, and psychological effects to perform a range of motion, strength, and endurance exercises. In a 10-week hydrotherapy program, 9 HSP patients were recruited [26]. All patients underwent individual exercise hydrotherapy sessions twice per week, with each session lasting 45 min and 2 small group sessions in a 10-week program. Spasticity was measured by MAS, and gait analysis parameters were analyzed in three different domains (i.e., spatiotemporal, kinetic, and kinematic). Spatiotemporal gait parameter analysis showed that treatment statistically improved walking speed (*p* = 0.03), while step length post-hydrotherapy improved, though not significantly (*p* = 0.07). Moreover, there was a reduction in the rotational range of motion at the hip (*p* < 0.01) and knee (*p* < 0.01). Kinematic and kinetic analyses showed that hydrotherapy increased hip internal rotation, allowing better foot clearance, probably due to an increase in compensatory strategies. The use of gait analysis is the main value of this study, but the uncontrolled design, the short-term duration of the intervention, and no mention of genetic diagnosis are the main limits of the study.

### Warming

In a quite recent study [27], 22 HSP patients (77% with a genetic diagnosis and/or family history of HSP, 17 patients with pure HSP), and 19 matched controls were enrolled to verify the effects of leg warming or cooling for 30 min on walking speed and local measures of neuromuscular functions. Walking speed was measured over a 10-m walkway, while other outcome measures like foot tap time, slow and fast stretches in order to explore muscle spasticity, and maximal isometric muscle strength at the ankle were also recorded. Tibial nerve conduction studies and motor-evoked potentials were even obtained. A single leg warming session ameliorated the dorsiflexor rate of force generation and decreased spasticity (stretch reflex size, *p* < 0.05) while cooling decreased walking speed (*p* < 0.05) and increased spasticity (*p* < 0.05).

A subsequent randomized crossover study by the same group evaluated the effects of superficial heating and insulation on motor performance of 21 HSP patients [28]. Bilateral gel-filled hot packs were applicated over the tibialis anterior and gastrocnemius muscles for 30 min and held in place by insulating wraps; in the no-insulation session, the insulating wraps were removed after 30-min while they were kept for an additional 30 min for the insulating session. Superficial heating improved walking speed (10 MWT, *p* < 0.0001, effect size 0.18) and foot tap time (*p* < 0.001, effect size 0.59) at 1 h after treatment. There was no further improvement in walking speed after leg insulation.

The quality of these studies is very low because intervention (warming) was not compared to standard therapy and included a single session with evaluation of short-term effects.

### Intensive physiotherapy

In a very small case series involving 2 HSP patients, an intensive physiotherapy program including stretching, strength, and functional exercises was performed for 60–90 min, 6 days per week, for 8 weeks (structured 8-week intensive rehabilitation program or SEIRP). Outcome measures were TUG, Functional Reach Test (FRT), 10 MWT, and 2 Minutes Walking Test (2 MWT) and were tested 4 and 8 weeks after the beginning of treatment. In all outcome measures, an improvement was registered [29]. The quality of this study is very low for the small sample size, the short follow-up period, and the absence of precise data about scale improvements.

## Interventional and surgical therapy

When spasticity is refractory to pharmacological and physical therapies, invasive approaches such as intrathecal baclofen, spinal cord stimulation, or selective ablative procedures can be attempted. For the purposes of this review, all papers addressing the effects of intrathecal baclofen were excluded because they configured a pharmacological approach. Only case reports evaluated the potential role of spinal cord stimulation and were therefore excluded from this review.

Kai et al. (2014) studied 4 patients with sporadic pure HSP (undefined genetic diagnosis) with severe lower limb spasticity (MAS score of at least 3) not responding to oral medications [30]. All these patients underwent a selective dorsal rhizotomy (SDR) after a laminectomy or laminotomy from L2 to S1. Intraoperative neurophysiological monitoring was used to prevent any damage directed to normal rootlets and preserve sphincter function. In a 2-year follow-up, there was a significant reduction of spasticity (Ashworth score, *p* < 0.01) and spasm frequency (lower extremity spasm frequency score, *p* < 0.01). Another retrospective study evaluated the effects of SDR on the spasticity of 4 patients with hereditary spastic paraparesis (age at surgery between 3 and 18). Only two patients with no genetic diagnosis had a clear-cut history of pure HSP and benefitted from the surgical procedure over time. The other two patients received a final genetic diagnosis of amyotrophic lateral sclerosis 2 (ALS2); in these latter two cases, there was no benefit, suggesting that SDR should be reserved for patients with a stable disease and a more predictable course [31].

The low quality of these two studies derives from very small cohorts, no definite genetic diagnosis, and no long-term assessment of SDR effects (more than 2 years) or risk assessment. Based on these two studies, SDR should be reserved for patients with pure HSP with a more predictable course and drug-resistant spasticity.

## Non-invasive stimulation techniques

Non-invasive stimulation techniques are known to modify neuronal activity by modulating synaptic plasticity and interfering with processes like long-term potentiation (LTP) or depression (LTD). Their advantages are represented by the low cost, portability, minimal side effects, and minimal awareness of the stimulation. These techniques can use magnetic fields to indirectly create electrical currents (i.e., magnetic stimulation) or directly deliver electrical currents (i.e., electrical stimulation).

Transcranial magnetic stimulation (TMS) warrants the electromagnetic induction of an electric field in a neuronal region and can be delivered as a single or repetitive pattern of application. Repetitive TMS (rTMS) can be either excitatory or inhibitory based on the frequency of stimulation, being respectively LTP or LTD mechanisms enhanced by high or low stimulation frequency [32]. Other rTMS protocols can produce short bursts of 50 Hz trains of stimuli at a rate in the theta range (5 Hz) continuously or intermittently in the so-called continuous and intermittent theta burst stimulation (i.e., cTBS and iTBS, respectively): cTBS suppresses neuronal excitability while iTBS increases it [33]. Recently, transspinal magnetic stimulation (TsMS) and root magnetic stimulations have been employed to reduce spasticity or motor impairment in Parkinson’s disease [34, 35], showing promising results in modulating spinal cord circuitry.

Low-amplitude currents can be delivered as direct or alternating currents over the scalp (transcranial electrical stimulation or tES) or the spine (transspinal direct current stimulation or tsDCS). These weak electrical currents are not able to induce action potentials but modulate neuronal firing rates and synaptic plasticity. In transcranial direct current stimulation (tDCS), constant-intensity currents are able to modulate synaptic and non-synaptic activity with an effect that depends on the polarity of the stimulation used: anodal and cathodal currents respectively enhance or inhibit neuronal excitability [36, 37]. tsDCS could work differently from tDCS because anodal currents could act on corticospinal descending pathways with an inhibitory effect, whereas cathodal stimulation could interfere with interneuronal networks [38, 39].

### Repetitive transcranial magnetic stimulation of the motor cortex

In the first study by Antczak et al. (2019), the effects of bilateral primary motor cortex high frequency (10 Hz) rTMS were evaluated in 14 patients with hereditary spastic paraplegia (9 patients with HSP, among them only 2 with a genetic diagnosis; the remaining 5 patients affected by adrenomyeloneuropathy) [40]. Most patients (7/14) used baclofen. This was a sham-controlled study consisting of real and sham 5 rTMS sessions, one per day for 5 consecutive days, with each subject undergoing real and sham stimulations. In every real stimulation session, 40 trains containing 75 stimuli lasting 7.5 s per hemisphere were delivered over the bilateral primary motor cortex by a double cone coil; for sham stimulation, the coil was held perpendicularly to the scalp. Assessment of the effects required clinical evaluation of walking speed (10 MWT) and other gait performance measures (TUG, strength by a dynamometer, and MAS). After real rTMS sessions, a reduction of proximal spasticity (*p* = 0.001) and an increase of proximal (*p* = 0.004) and distal (*p* = 0.041) muscular strength were observed immediately after the last stimulation session. Only proximal spasticity reduction (*p* = 0.018) was observed at the follow-up visit (2 weeks after the stimulation). There was no significant effect on walking speed (*p* = 0.07). This study describes rTMS as a potential tool to treat motor symptoms in HSP patients. However, no precise genetic diagnosis in the HSP sample, the limited number of sessions (only five for 1 week), the short duration of follow-up (2 weeks), the lack of neurophysiological assessment after intervention, and the unblinded evaluation of datasets are the main limitations of this study. Moreover, safety issues—seizures occurred in 1 patient—suggest adopting precautions when using rTMS in HSP patients.

In another study, 8 patients were randomly assigned to receive real (*n* = 4) or sham (*n* = 4) high-frequency rTMS over the vertex. rTMS was performed during five daily sessions at a fixed frequency of 5 Hz by an eight-figure-shaped coil [41]. Every session consisted of 5 trains lasting 1 min (1500 pulses per session), while sham stimulation was performed by placing the coil perpendicular to the scalp. Outcome measures were MAS, Fugl-Meyer Assessment (FMA-LE), 10 MWT, and SF-36. The authors observed a reduction of lower limb spasticity, as assessed by the MAS, immediately after five sessions of rTMS (*p* = 0.019) and 1 month after the stimulation sessions (*p* = 0.048); also in this study, rTMS was not effective in improving other outcomes such as walking speed and quality of life. In this study, no adverse effect was reported after rTMS.

Despite the small sample size, both studies using TMS were well designed and suggest using rTMS to treat some motor symptoms in HSP. Comparative studies are needed in order to optimize therapy which can be very different in frequency of stimulation, type of coil, number of stimulation sessions, and duration of follow-up.

### Transspinal magnetic stimulation

In the only open-label pilot available trial, the authors enrolled 3 patients with a definite genetic diagnosis of HSP [42]. All patients underwent a single session of transspinal magnetic stimulation (TsMS) at the level of the second thoracic vertebra by a continuous theta burst session of a hundred three-pulse bursts at 50 Hz repeated after a 90-min interval using a figure-eight coil and at 100% of the motor threshold. In other disease models, TsMS could have different effects on motor symptoms and improve neurogenic bladder and bowel disorders. In this exploratory trial, neurophysiological outcomes were tested, and more precisely, the H-reflex recovery curve to paired stimuli was measured. TsMS was effective in reducing the hyperexcitability of the stretch reflex in this small sample of HSP patients, suggesting that this technique could modulate spinal cord hyperexcitability. The quality of this trial is very low because only a single session of TsMS was performed, a very small cohort of patients was recruited, and only immediate and short-term effects of TsMS were tested; moreover, no clinical assessment of motor and non-motor impairment was made.

### Magnetic root stimulation

In an open-label trial, 15 patients with different types of spinal lesions (only 2 patients had a clear-cut history of HSP, 6 with multiple sclerosis, 1 with vasculitis, 1 with transverse myelitis, and 4 with an unknown cause) and 16 healthy controls underwent a single session of unilateral magnetic stimulation at the level of the L3-L4 vertebra [43]. A 90-mm-circular coil was used to deliver 20-Hz repetitive stimulation (2000 pulses) at 120% of lower limb contraction. Only patients displayed a reduction of spastic tone as assessed by the MAS score and the peak velocity of the first swing of the pendulum test 4–24 h (*p* < 0.008) after stimulation. These effects were observed ipsilaterally but also contralaterally to stimulation, subtending a spinal modulatory effect of magnetic lumbar stimulation. No adverse effect was reported. This study was included in this review because two cases of HSPs were treated, but the quality is very low for several reasons: the enrolled patients exhibited very different pathophysiological processes responsible for spasticity; there was no specific subanalysis of different clinical populations; only a single session of magnetic stimulation was performed; no sham-controlled session was obtained; only short-term effects were evaluated; and no neurophysiological measurement of spinal excitability was obtained.

### Spinal direct current stimulation

In the only trial using tsDCS in a crossover design, 11 HSP patients underwent real or sham tsDCS over the spinous process of the tenth thoracic vertebra (reference electrode over the right shoulder) at an intensity of 2 mA for 20 min [44]. Each session lasted 5 days a week (20 min twice a day). Outcome measures were explored at the end of the stimulation week (T1), after 1 week (T2), 1 month (T3), and 2 months (T4). Anodal tsDCS improved spasticity as assessed by the MAS up to 2 months after the end of stimulation (T1 *p* = 0.0137; T4 *p* = 0.0244), especially for knee extension (T4 *p* = 0.0039) and hip flexion (T4 *p* = 0.016), with no modification of neurophysiological measures (F-waves, H-waves, and motor-evoked potentials). These results could support the hypothesis that tsDCS can induce pre-synaptic inhibition and post-activation depression, interfering with the maladaptive spinal hyperexcitability responsible for spasticity. Even if this study shows promising results, the small number of patients, the clinical heterogeneity of patients enrolled, and the lack of evaluation of non-motor features make the results of this study exploratory and need further confirmation in future studies.

## Discussion

Although advances have been made in the genetic and pathogenetic knowledge of HSPs, symptomatic therapy is still the mainstay of treatment due to the lack of genetic therapy and the unsatisfying response to conventional therapy.

The studies included in this systematic review show important limitations, especially for methodology, population, intervention, and outcome measures, making a meta-analysis not feasible. Moreover, most studies addressed motor involvement in HSP with a mention of spasticity, but no study addressed the issue of sensory and urinary symptoms.

The present systematic review considered 14 studies exploring the role of non-pharmacological management of HSP: 10 studies were class IV studies (case series, retrospective studies, and uncontrolled studies) and 4 were class III studies (1 exploring the effect of warming combined with insulation and 3 sham-controlled trials investigating non-invasive stimulation techniques, 2 of them with a cross-over design). Table 1 summarizes all the studies considered.

**Table 1:** Overview of the studies included

Author (year) [reference]	Title of paper	Genetic type of HSP	No. of subjects	Treatment	Outcome measures	Type of study
Physical therapy						
Marsden et al. (2011) [23]	The effects of functional electrical stimulation on walking in hereditary and spontaneous spastic paraparesis	Not specified	11 patients (6 with a family history)	Bilateral stimulation of the common peroneal nerve (BICP) (*n* = 8) or preferred pattern of stimulation (PREF) (*n* = 3)	Dorsiflexor torque, range of movement of dorsiflexion and degree of toe clearance while walking, 10-Meter Walking Test (10 MWT) for walking speed, Physiological Cost Index (PCI)	Uncontrolled prospective study
Bertolucci et al. (2015) [24]	Robotic gait training improves motor skills and quality of life in hereditary spastic paraplegia	SPG4 – SPG5 – SPG7 – SPG11	13 patients (SPG4 *n* = 5; SPG5 *n* = 1; SPG7 *n* = 6; SPG11 *n* = 1)	Lokomat, for three weekly sessions, for a 6-week long period	Berg Balance Scale (BBS), the Timed Up and Go Test (TUG), 6 Minutes Walking Test (6 MWT), 10 MWT, the Modified Ashworth Scale (MAS), PCI, Hospital Anxiety and Depression Scale, and the SF-36 scale	Uncontrolled prospective study
Zhang et al. (2014) [25]	The effect of hydrotherapy treatment on gait characteristics of hereditary spastic paraparesis patients	Not specified	9 patients	10-week hydrotherapy program	MAS and gait analysis in 3 domains (spatiotemporal, kinematic, and kinetic)	Uncontrolled prospective study
Denton et al. (2016) [26]	Superficial warming and cooling of the leg affects walking speed and neuromuscular impairments in people with spastic paraparesis	SPG3a – SPG4 – SPG10 – not specified	22 patients (8 received a genetic diagnosis, SPG3a *n* = 1; SPG4 *n* = 6; SPG10 *n* = 1); 19 matched healthy controls	Single session of cooling or warming of the leg for 30 min	10 MWT, foot tap time, slow and fast stretches, maximal isometric muscle strength at the ankle, tibial nerve conduction studies, and motor-evoked potentials	Controlled prospective study
Denton et al. (2018) [27]	Effects of superficial heating and insulation on walking speed in people with hereditary and spontaneous spastic paraparesis: a randomised crossover study	Not specified	21 patients	Superficial heating of the leg combined or not with insulation	10 MWT and foot tap time	Randomized crossover study
Samuel et al. (2013) [28]	Physical therapy interventions for the patients with hereditary spastic paraparesis—an exploratory case reports	Not specified	2 patients	Structured 8-week intensive rehabilitation program or SEIRP, including stretching, strength, and functional exercises for 60–90 min per day, 6 days a week, 8 weeks	TUG, Functional Reach Test (FRT), 10 MWT, and 2-min walking test (2 MWT)	Case series
Interventional and surgical therapy						
Kai et al. (2014) [29]	Long-term results of selective dorsal rhizotomy for hereditary spastic paraparesis	Not specified	4 patients	Selective dorsal rhizotomy (SDR) under neurophysiological monitoring	Ashworth score and spasms frequency score	Case series
Sharma et al. (2016) [30]	Selective dorsal rhizotomy for hereditary spastic paraparesis in children	Not specified	2 patients	SDR	MAS sum score, Gross Motor Function Measure Score Sheet (GMFM-88), and muscle strength	Retrospective study
Non-invasive stimulation techniques						
Antczak et al. (2019) [39]	The effect of repetitive transcranial magnetic stimulation on motor symptoms in hereditary spastic paraplegia	SPG3a – SPG7 – not specified	9 patients (SPG3a *n* = 1; SPG7 *n* = 1)	5 daily sessions of 10-Hz repetitive transcranial magnetic stimulation (rTMS) over the bilateral primary motor cortex of lower limb muscles vs. sham rTMS over the same area (40 trains lasting 7.5 s per hemisphere per session)	10 MWT, TUG, muscle strength measured by a dynamometer, MAS	Randomized cross-over controlled trial
Bastani et al. (2021) [40]	A randomized controlled trial of the effect of repetitive transcranial magnetic stimulation of the motor cortex on lower extremity spasticity in hereditary spastic paraplegia	Not specified	8 patients	Active or sham rTMS over the vertex (5 daily sessions of 5-Hz rTMS, 5 trains per session, each lasting 1 min)	MAS, Fugl-Meyer Assessment (FMA-LE), 10 MWT, SF-36	Randomized controlled trial
Carra et al. (2022) [41]	Controversies and clinical applications of non-invasive transspinal magnetic stimulation: a critical review and exploratory trial in hereditary spastic paraplegia	SPG4 – SPG33	3 patients (SPG4 *n* = 1; SPG33 *n* = 2)	Transpinal magnetic stimulation (TsMS) at the level of the second thoracic vertebra by continuous theta burst session of a hundred three-pulse bursts at 50 Hz repeated after a 90-min interval	H-reflex recovery curve to paired stimuli	Open-label pilot uncontrolled trial
Krause et al. (2004) [42]	Lumbar repetitive magnetic stimulation reduces spastic tone increase of the lower limbs	Not specified	2 patients with familial spasticity among 15 spinal lesion patients; 10 matched controls	Repetitive magnetic stimulation over unilateral L3-L4 roots (20 Hz, 2000 pulses, 1 session)	MAS, pendulum test	Open-label trial
Ardolino et al. (2021) [43]	Spinal direct current stimulation (tsDCS) in hereditary spastic paraplegias (HSP): a sham controlled crossover study	SPG3a – SPG4 – SPG7 – SPG15	11 patients (SPG3a *n* = 1; SPG4 *n* = 8; SPG7 *n* = 1; SPG15 *n* = 1)	Five daily sessions of anodal transpinal direct current stimulation (tsDCS) over the thoracic spinal cord (T10-T12) vs. sham tsDCS (intensity current: 2 mA, duration of stimulation: 20 min)	Motor-evoked potentials (MEPs), the H-reflex, F-waves, Ashworth scale, the Five Minute Walking Test, and the Spastic Paraplegia Rating Scale (SPRS)	Randomized cross-over controlled trial

*10 MWT* 10 Meter Walking Test, *2 MWT* 2 Minutes Walking Test, *6 MWT* 6 Minutes Walking Test, *BBS* Berg Balance Scale, *BICP* bilateral stimulation of the common peroneal nerve, *FMA-LE* Fugl-Meyer Assessment, *FRT* Functional Reach Test, *GMFM-88* Gross Motor Function Measure Score Sheet, *MAS* Modified Ashworth Scale, *MEPs* motor-evoked potentials, *PCI* Physiological Cost Index, *rTMS* repetitive transcranial magnetic stimulation, *SDR* selective dorsal rhizotomy, *SF-36* Short Form Health Survey 36, *SPRS* Spastic Paraplegia Rating Scale, *tsDCS* transpinal direct current stimulation, *TsMS* transpinal magnetic stimulation, *TUG* Timed Up and Go Test

Most studies showed very low quality because of the very small sample size. Not all patients have received a genetic diagnosis, and this is relevant for several reasons. Firstly, results obtained from patients without a genetic confirmation cannot be compared to those obtained from patients with a genetic diagnosis. Secondly, although natural history studies are lacking, it is known that pure and complicated HSPs may have different disease courses and are clinically heterogenous, requiring different therapeutic approaches.

Rehabilitative protocols in HSPs use very heterogenous timing and follow-up. Accordingly, a lot of studies exploring the effect of physiotherapy were excluded from this review because they were only case reports or studies with a combined pharmacological and physical approach [18, 19]. None of the selected studies evaluated a combined approach of non-pharmacological techniques to treat motor disability. Further studies should address the issue of combined non-pharmacological approaches to warrant better outcomes in this patient population.

The outcome measures in all the included studies were rather heterogenous. Walking speed was often measured using the 6 MWT or the 10 MWT. Gait analysis was conducted in only one study [26]. Psychological status and quality of life were considered in some studies. In most studies, the MAS was used to quantify lower limb rigidity even if some studies used different methods to detect it [27]. The use of MAS has several disadvantages to take into account: it is a subjective scale, it does not measure muscle strength, it does not consider the level of pain and discomfort, it is not really accurate in evaluating mild spasticity, and it does not consider other features of the UMN syndrome such as clonus, spasms, and secondary muscle abnormalities. Moreover, evaluation scales used to track results are not always effective for identifying clinically significant changes, and none of the included studies evaluated Patient-Reported Outcome Measures (PROMs). Further studies are needed to overcome the abovementioned issues promoting the use of PROMs and a broader assessment of spasticity which should include muscle strength evaluation, neurophysiological examinations (e.g., H-reflex recovery curves), MAS scale, spasm frequency, and viscoelastic changes involving spastic muscles (e.g., elastosonography).

Moreover, sensory impairment has never been the objective of treatment in HSP patients. Indeed, altered proprioception due to posterior column deterioration is responsible for the delayed postural response and consequentially contributes to the increased risk of falls in HSP [45]. This aspect should be further explored in future studies.

**Fig. 2:**
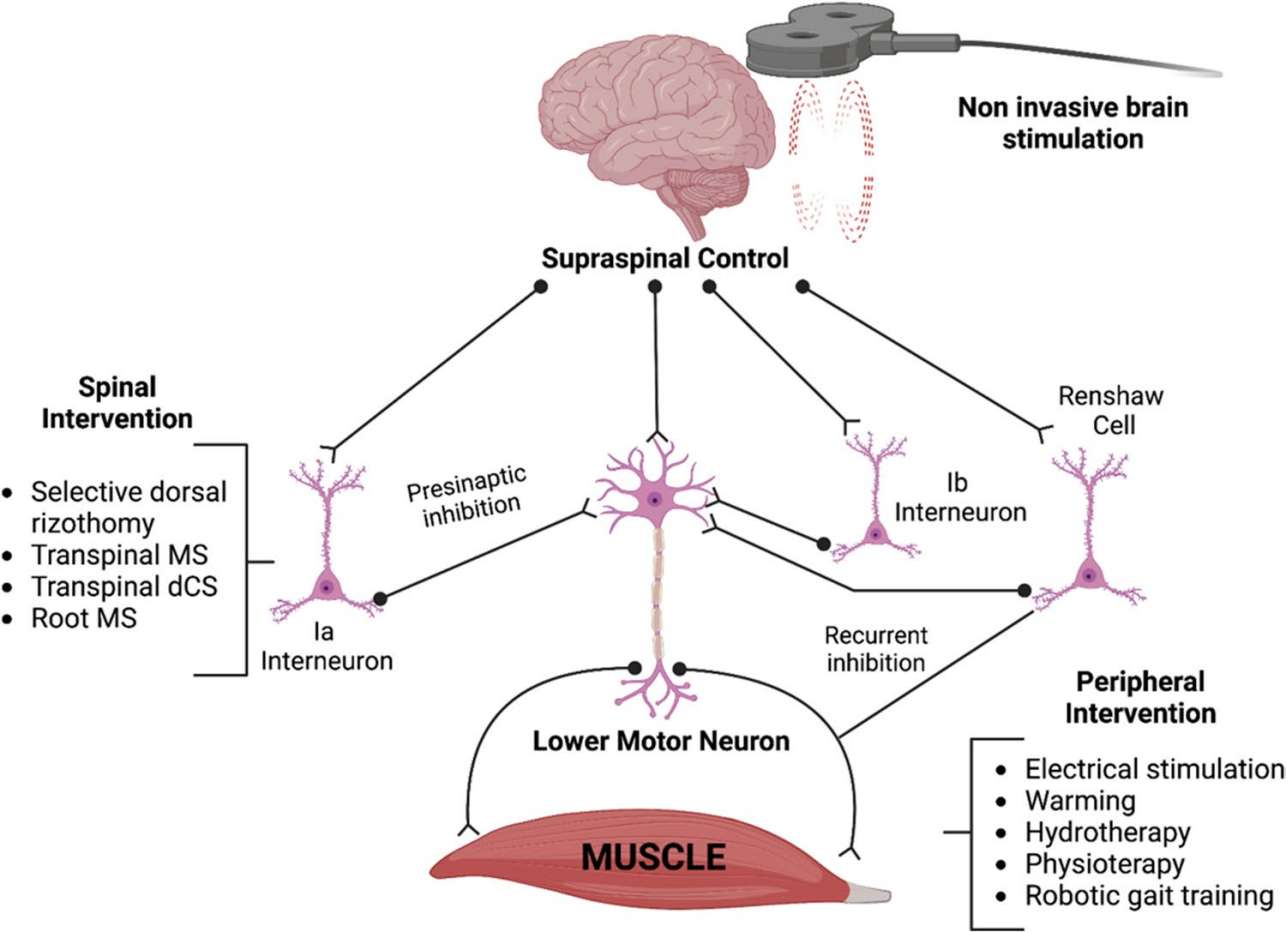
Summary of non-pharmacological interventions in HSP-related spasticity. Abbreviations: dCS, direct current stimulation; MS, magnetic stimulation

Despite the mentioned limitations, new pathophysiological insights into spasticity can be obtained from this review. Even in HSP, spasticity is not a static process, and several plastic changes can occur both in the central nervous system (from the motor cortex to the spine) and at the level of the muscle. A summary of non-pharmacological interventions for UMN syndrome already described in HSP is described in Fig. 2.

Spasticity is primarily due to the loss of inhibitory influences on the stretch reflex, normally warranted by the dorsal reticulospinal tract [46]. Post-synaptic inhibition is the consequence of reciprocal Ia inhibition [47], Ib inhibition [48], and recurrent inhibition [49], but pre-synaptic Ia inhibition also exists [50], ensuring the reduction of neurotransmitter release in the synaptic cleft between primary afferent Ia fibers and motor neurons. Moreover, plastic changes take place in the UMN syndrome because of spinal alpha motor neuron denervation (“denervation hypersensitivity”). Denervated alpha motor neurons become hyperexcitable even because they start to release growth factors, promoting local sprouting from interneurons and leading to the formation of new somatic synapses which occupy the space left empty by the missing descending fibers [51]. Increasing motor cortex output to alpha spinal motor neurons by primary motor cortex rTMS can help reduce spasticity and improve muscle strength because it promotes both pre-synaptic and post-synaptic inhibition at the spinal level. These effects lasted more than 2 weeks after stimulation sessions, corroborating previous results in TMS studies showing that rTMS can remodel neural circuits, enhance brain-derived growth factor (BDNF) signaling, and upregulate N-methyl-D-aspartate (NMDA) receptors [52]. New techniques such as TsMS and tsDCS have shown promising results in treating HSP spasticity, as these two kinds of stimulation are able to modulate spinal circuitry. As for TsMS, parts of the effects are due to peripheral root stimulation and Ia afferent stimulation that can reduce spinal motor neuron hyperexcitability, while tsDCS can enhance descending inhibitory influences at a spinal level. All these results suggest that spasticity therapy should be targeted in combined protocols involving brain and spine modulation in HSP but also in other diseases.

## Conclusion

The management of clinical manifestations in HSPs is challenging, even though the search for an effective treatment is still lagging. We cannot exclude that our search was incomplete and overlooked signification information coming from case reports and articles not written in English.

Pharmacological symptomatic treatment is still the cornerstone of HSP management, but a multidisciplinary approach can offer several advantages in order to ameliorate motor symptoms, in addition to improving the quality of life and psychological status of patients. A greater effort should be made to obtain larger samples, and this review confirms the need for multicenter studies. Studies exploring the combined effect of non-pharmacological treatment using different targets (e.g., motor cortex, spinal circuitries, and muscles) should be performed in order to obtain better outcomes.
